# Obituary

**Published:** 1847-05

**Authors:** 


*Obituary.*—"April 30th. With deep regret we are compelled to insert the following announcement of the death of the amiable and accomplished Prof. of Practice in the University. His health has long been feeble, but the immediate cause of his death we suppose to have been Pneumonia. In a future number we hope to furnish a sketch of the life of this distinguished teacher.

"At his residence in Ninth street, last evening, John Revere, M. D., Professor in the Medical School of the University of New York, aged 60 years."—*N. Y. Annalist.*

At his residence in Marietta, Ohio, on the 2d inst., Hon. John Cotton, M. D.

In New York city, April 18th, of typhoid fever, Samuel W, Farrar, M. D., assistant Physician to the Bloomingdale Asylum.

At Farmsville, Ohio, on the 17th of March, Dr. Jos. Evans, aged 78 years.

